# A novel method for measurement of the occipital-cervical distance via the occiput-C4 distance

**DOI:** 10.1186/s12891-020-03398-9

**Published:** 2020-06-15

**Authors:** Chao Tang, Sheng Yang, Ye Hui Liao, Qiang Tang, Fei Ma, Qing Wang, De Jun Zhong

**Affiliations:** Department of Spine Surgery, Affiliated Hospital of Southwest Medical University, No. 25 Taiping Street, Luzhou City, 646000 China

**Keywords:** Occiput-C4 distance, Occipitocervical distance, C4 vertebral body, McGregor’s line

## Abstract

**Background:**

The aim of the present study was to describe and measure the occipital-cervical distance by a novel method utilizing the occiput-C4 distance (OC4D) in normal subjects, as a proposed tool to guide restoration of vertical dislocations of the occipitocervical region in patients with basilar invaginations and for performing standardized testing of occipitocervical constructs.

**Methods:**

We analyzed neutral, flexion, and extension lateral cervical spine radiographs of 150 asymptomatic subjects (73 males and 77 females) that were judged to be normal. The mean age of the included asymptomatic subjects was 48.0 ± 8.4 years old (range 20–69 years old; 48.4 ± 10.2 years old for males and 47.6 ± 6.4 years old for females). The OC4D was defined as the shortest distance from the center of the C4 vertebral body to the McGregor’s line. Occipitocervical distances (OCDs) were measured and analyzed its correlation with OC4Ds. Two spine surgeons each performed three measurements of the OC4D and OCD from each asymptomatic subject, from which our reported average values were derived. The height, weight, and body mass index (BMI) of each subject were recorded and analyzed for their correlations with the OC4D and OCD.

**Results:**

The OC4Ds from neutral, flexion, and extension lateral cervical spine radiographs were 69.0 ± 6.9, 68.9 ± 6.8, and 68.1 ± 6.9 mm, respectively. There was no significant difference in the OC4D values among neutral, flexion, and extension lateral cervical spine radiographs (*P* >  0.05). The neutral, flexion, and extension OCDs were 23.0 ± 4.8, 27.6 ± 6.0, and 13.8 ± 4.7 mm, respectively. In particular, the neutral OCD was significantly different from those in flexion and extension lateral cervical spine radiographs (*P* <  0.001). There was no significant correlation between OC4D and OCD in neutral, flexion, and extension (*P* >  0.05 for all). There were positive correlations between OC4D and height, as well as OC4D and weight, in neutral, flexion, and extension lateral cervical spine radiographs (*P* <  0.001 for all). Furthermore, the intra-class correlation coefficients for inter- and intra-observer reliabilities of OC4Ds in neutral, flexion, and extension lateral cervical spine radiographs were significantly higher than those for OCDs (*P* <  0.001).

**Conclusions:**

The OC4D represents a novel measurement for estimating the occipital-cervical distance that is not affected by changes in neutral, flexion, and extension positions. Hence, the OC4D may serve as a valuable parameter and intra-operative tool to guide vertical restoration during occipitocervical fusion (OCF) for patients with altered occiput-cervical anatomy.

## Background

Craniocervical joint instability caused by a congenital anomaly (e.g., basilar invagination), trauma, inflammatory disease, or a tumor may be an indication for occipitocervical fusion (OCF) [1]. During occipitocervical fixation and fusion, it is important to confirm that the occiput remains in a neutral balanced position in relation to the cervical spine. Previous studies have tended to focus on the effect of occipitocervical angle fixation on postoperative dyspnea/dysphagia and lower cervical spine degeneration [2, 3]. However, few studies have focused on the relationship between the distance of occipital-cervical vertical reduction and lower cranial nerve palsy following vertical over-distraction. Therefore, in the context of our present study, we considered that a normal occipital-cervical distance is likely important for avoiding over-distraction injuries to the cranial nerves and spinal cord during OCF. Previously, the occipital-cervical distance has been obtained by measuring the shortest distance from the most superior aspect of the C2 spinous process to the occipital protuberance [4]; however, this measurement method is significantly affected by even minimal head rotation. The C4 vertebral body, with a landmark at the mid-cervical level, is less affected by upper cervical spine motion/rotation, which makes it a more effective and versatile landmark for defining the occipitocervical neutral position during fusion surgery [5].

In the present study, we introduced and evaluated the occiput-C4 distance (OC4D) measurement method using lateral cervical spine radiographs from asymptomatic subjects. Additionally, we measured and compared the OC4Ds in different cervical positions and obtained normal value ranges, and further analyzed the correlation of OC4Ds with weight, height, and body mass index (BMI) in order to more comprehensively and accurately define the occipitocervical neutral position. Our findings may support the future use of our practical OC4D measurement method and its reference values for defining occiput-cervical vertical reduction during OCF.

## Methods

### Subjects

In this study, we included 150 sagittal-balanced cervical spine lateral radiographs, which were interpreted to be normal by two spine surgeons (i.e., absence of fractures or dislocations, absence of deformities, absence of severe osteophyte formation, absence of destruction of the vertebrae, and absence of spondylosis), from a radiographic database at the Affiliated Hospital of Southwest Medical University (China). Sagittal-balanced asymptomatic subjects were evaluated via both whole spine radiographs and cervical radiographs.

### Study sample

The study population consisted of 73 males and 77 females with an average age of 48.0 ± 8.4 years (range 20–69 years; 48.4 ± 10.2 years for males and 47.6 ± 6.4 years for females). All subjects in the present study were from Southwest China, with an average height of 162.4 ± 8.2 cm (males: 168.9 ± 10.4 cm, range: 156–182 cm; females: 159.7 ± 6.8 cm, range: 148–175 cm) and average weight of 62.4 ± 10.3 kg (males: 64.3 ± 12.9 kg, range: 49–92 kg; females: 58.4 ± 9.6 kg, range: 42–85 kg). Of the 150 participants included in the study, 79.3% (119/150) were of Han ethnicity. The proportions of the Yi, Zhuang, Manchu, Hui, Miao, and Mongolian ethnicities were 6.0% (9/150), 4.7% (7/150), 4.7% (7/150), 2.0% (3/150), 2.0% (3/150), and 1.3% (2/150), respectively. Approval for this study was obtained from the Ethics Committee of the Affiliated Hospital of Southwest Medical University (China).

### Measurements and procedure of OC4D and OCD

**Fig. 1:**
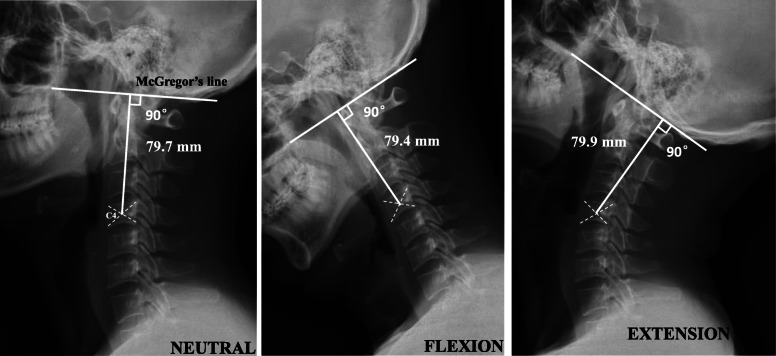
A novel measurement method for estimating the occipital-cervical distance via the occiput-C4 distance (OC4D) in representative neutral, flexion, and extension positions. The OC4D was defined as the shortest distance from the center of the C4 vertebral body to the McGregor’s line

**Fig. 2:**
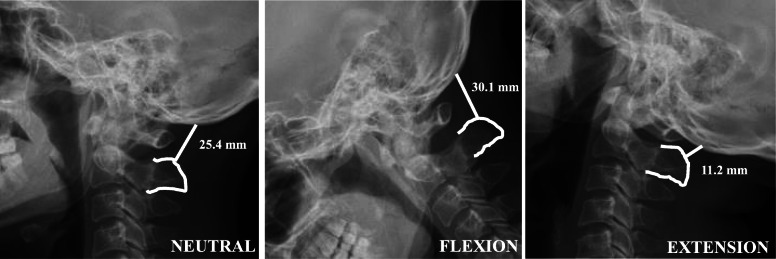
Differences in the measured occipitocervical distance (OCD) due to cervical positions in neutral, flexion, and extension positions. OCD was defined as the shortest distance from the most superior aspect of the C2 spinous process to the occipital protuberance

Standard lateral cervical radiographs were obtained from each subject. The X-ray tube was centered at C4, and the radiographs were taken from a distance of 2 m from the subject’s left side. Analysis of lateral neutral, flexion, and extension radiographs was performed using our novel OC4D measurement method. The OC4D was defined as the shortest distance from the center of the C4 vertebral body to the McGregor’s line (Fig. 1). In contrast, the occipitocervical distance (OCD) was defined as the shortest distance between the most superior aspect of the C2 spinous process and the occipital protuberance (Fig. 2) [4]. Two spine surgeons calculated and documented the OC4D and OCD in each of the tested cervical radiographical positions in a blinded manner. These parameters were measured by both observers on three occasions over an interval of at least 2 weeks.

To evaluate errors in these measurements, the differences among the means of the first, second, and third tracings for each of the variables were tested using paired t tests. No statistically significant differences were found. Therefore, the average measured values were used for subsequent comparisons. The height, weight, and BMI of each subject were recorded by the co-authors in this study, and BMI was calculated as follows:

$$ \mathrm{BMI}=\mathrm{Weight}\ \left(\mathrm{kg}\right)/\mathrm{Height}\ {\left(\mathrm{m}\right)}^2 $$

### Statistical analysis

SPSS software version 19.0 (SPSS Inc., Chicago, IL, USA) was used for all statistical analyses. Comparisons of the mean differences between groups that have been split on two independent variables (factor 1: radiographical position; factor 2: gender) were performed using Two-Way ANOVA and *post-hoc* tests. Pearson’s coefficient was used to analyze correlation between OC4D and OCD, and correlations of OC4D/OCD with height, weight, and BMI. Inter- and intra-observer reliabilities of OC4Ds and OCDs were assessed by calculating intra-class and inter-class correlation coefficients (ICCs). The calculated ICCs were interpreted according to the following standard convention: 0.90–1.0, excellent agreement; 0.70–0.89, good agreement; 0.50–0.69, fair/moderate agreement; 0.25–0.49, low agreement; and ≤ 0.24, poor or absent agreement [6]. Comparison of the ICC values was performed using the Z test. Data are expressed as the mean ± standard deviation of the mean, and differences between distributions were considered statistically significant at *P* <  0.05. All reliability estimates are presented with a 95% confidence interval (CI).

## Results

### Measurements of the OC4D and OCD

The mean value of the OC4D in the neutral position was 69.0 ± 6.9 mm, which was not significantly different from the OC4D measured in flexion (68.9 ± 6.8 mm) or extension (68.1 ± 6.9 mm; *P* >  0.05). In contrast, the mean neutral, flexion, and extension OCDs were 23.0 ± 4.8 mm, 27.6 ± 6.0 mm and 13.8 ± 4.7 mm, respectively; the neutral OCD was significantly greater than that in flexion, but was significantly smaller than that in extension (*P* <  0.001; Table 1).

**Table 1:** Measurements of the OC4D and OCD in neutral, flexion, and extension positions

	Neutral (*n* = 150)	Flexion (*n* = 150)	Extension (*n* = 150)	Group difference (*P* value)
**OC4D** (mm)	69.0 ± 6.9	68.9 ± 6.8	68.1 ± 6.9	0.302
**OCD** (mm)	23.0 ± 4.8	27.6 ± 6.0	13.8 ± 4.7	F > N > E (P < 0.05)

Values represent the mean ± standard deviation

*OC4D* Occiput-C4 distance, *OCD* Occipitocervical distance

For the OC4Ds, the values in males were significantly higher than those in females in neutral, flexion, and extension positions (*P* <  0.001 for all). However, there was no significant gender differences for OCDs in neutral, flexion, or extension positions between males and females (*P* >  0.05 for all; Table 2).

**Table 2:** Gender differences of the OC4D and OCD in neutral, flexion, and extension positions

	Male (*n* = 73)	Female (*n* = 77)	***P*** value
**OC4D** (mm)			
Neutral	73.0 ± 6.4	65.1 ± 4.8	< 0.001
Flexion	72.0 ± 6.6	64.4 ± 4.9	< 0.001
Extension	72.9 ± 6.3	65.1 ± 4.9	< 0.001
**OCD** (mm)			
Neutral	22.4 ± 5.0	23.6 ± 4.7	> 0.05
Flexion	26.4 ± 4.7	28.8 ± 7.0	> 0.05
Extension	13.1 ± 4.6	14.5 ± 4.7	> 0.05

Values represent the mean ± standard deviation

*OC4D* Occiput-C4 distance, *OCD* Occipitocervical distance

### Correlations between OC4D and OCD

Pearson’s correlation coefficients showed that OC4D were weak negatively correlated with OCD (*r* = − 0.164 in neutral, *r* = − 0.171 in flexion, and *r* = − 0.038 in extension), but there was no significant difference (*P* >  0.05 for all) (Table 3).

**Table 3:** Bivariate correlations between OC4D and OCD

	Neutral	Flexion	Extension
Pearson correlation coefficient	− 0.164	− 0.171	− 0.038
*P* value	0.068	0.058	0.677

*OC4D* Occiput-C4 distance, *OCD* Occipitocervical distance

### Correlations of OC4Ds/OCDs with height, weight, and BMI

Pearson’s correlation coefficients showed that OC4Ds were significantly positively correlated with height (*r* = 0.707 in neutral, *r* = 707 in flexion, and *r* = 0.666 in extension; *P* <  0.001 for all), and there was also a moderate correlation between OC4D and weight (*r* = 0.541 in neutral, *r* = 0.541 in flexion, and *r* = 0.505 in extension; *P* <  0.001 for all). In contrast, there was no significant correlation between OC4D and BMI (*P* >  0.05; Table 4).

**Table 4:** Bivariate correlations of OC4Ds with height, weight, and BMI

	Height	Weight	BMI
**Neutral**			
Pearson correlation coefficient	0.707	0.541	0.131
*P* value	< 0.001	< 0.001	<0.176
**Flexion**			
Pearson correlation coefficient	0.707	0.541	0.126
*P* value	< 0.001	< 0.001	0.098
**Extension**			
Pearson correlation coefficient	0.666	0.505	0.111
*P* value	< 0.001	< 0.001	0.145

*OC4D* Occiput-C4 distance, *BMI* Body mass index

For OCDs, there was a weak but significant correlation with height in each tested position (*r* = 0.284 in neutral, *r* = 0.239 in flexion, and *r* = 0.215 in extension; *P* <  0.05 for all). However, there was no significant correlation between OCD and weight, or between OCD and BMI, in neutral, flexion, and extension positions (Table 5).

**Table 5:** Bivariate correlations of OCDs with height, weight, and BMI

	Height	Weight	BMI
**Neutral**			
Pearson correlation coefficient	0.284	0.100	−0.170
*P* value	< 0.001	0.27	0.059
**Flexion**			
Pearson correlation coefficient	0.239	0.036	−0.180
*P* value	0.007	0.688	0.066
**Extension**			
Pearson correlation coefficient	0.215	0.055	−0.133
*P* value	0.017	0.544	0.142

*OCD* Occipitocervical distance, *BMI* Body mass index

### Inter- and intra-observer agreements

The inter-observer reliabilities of OC4Ds were found to have ICCs of 0.945, 0.953, and 0.961 in neutral, flexion, and extension positions, respectively. Additionally, the ICC values in terms of intra-observer reliabilities for OC4Ds were 0.981, 0.972, and 0.968 in neutral, flexion, and extension positions, respectively (Table 6). Hence, all ICC values for both inter-and intra-observer reliabilities of OC4Ds in neutral, flexion, and extension positions were excellent, as they were all above 0.93. No significant difference was found between the measurements made by a single observer and those made by different observers. Finally, ICCs of inter- and intra-observer reliabilities for OC4D were significantly higher than those for OCD in each tested position (*P* <  0.05; Table 6).

**Table 6:** Inter- and intra-observer reliabilities of OC4Ds and OCDs

		OC4D		OCD			*P* value*
	ICC	Std error	95% CI	ICC	Std error	95% CI	
**Neutral**							
inter-observer reliability	0.945	0.185	0.921–0.965	0.785	0.254	0.689–0.814	< 0.05
intra-observer reliability	0.981	0.124	0.941–0.988	0.832	0.239	0.812–0.841	< 0.05
**Extension**							
inter-observer reliability	0.953	0.173	0.942–0.971	0.654	0.312	0.638–0.664	< 0.001
intra-observer reliability	0.972	0.142	0.953–0.985	0.671	0.301	0.651–0.685	< 0.001
**Flexion**							
inter-observer reliability	0.961	0.156	0.948–0.987	0.681	0.289	0.678–0.688	< 0.05
intra-observer reliability	0.968	0.151	0.955–0.989	0.695	0.262	0.684–0.715	< 0.001

*OC4D* Occiput-C4 distance, *OCD* Occipitocervical distance

*Comparison of the ICC values between OC4D and OCD using the Z test

## Discussion

In this study, we determined the reference values for estimating the occipital-cervical distance in neutral, flexion, and extension positions via our novel OC4D measurement method, which may provide a comprehensive and accurate estimation for vertical reduction of the occiput-cervical region during OCF. Importantly, the OC4D—as a simple, convenient, and highly reliable measurement of occiput-cervical distance—is not occluded by implants. More importantly, our present study revealed that the OC4D is not affected by changes in neutral, flexion, and extension cervical positions.

Conceptually, the occipitocervical neutral position is the functional and balanced position of the head atop the cervical spine. We considered that patients should have a normal occipitocervical angle and occiput-cervical distance in this neutral position. Sherekar et al. [7] measured the occipito-C2 angle in 518 asymptomatic volunteers (261 male and 257 female subjects), and obtained values of 14.66 ± 9.5° in males and 15.59 ± 8.26° in females. Many researchers have reported that non-normal occipitocervical angles lead to poor postoperative fusion, and even severe dysphagia and/or dyspnea during OCF [3, 8–10]. However, it remains unknown whether dysphagia and/or dyspnea are mostly due to mechanical airway obstruction caused by a non-normal occipitocervical angle. We believe that surgeons should pay more attention to the lower cranial nerve stretch airway obstruction caused by over-distraction of the occiput-cervical vertical distance. Shigeto et al. reported that the mechanism of dysphagia is not simply associated with the O-C2 angle, but that it also involves global craniocervical alignment in an individual patient, including the occiput-cervical distance [11]. Wang et al. reported that performing OCF in the over-distraction position to treat vertical atlantoaxial dislocation may caudally displace the brainstem relative to the cranial base, resulting in traction injury to the 9th, 10th, and 11th lower cranial nerves [12].

**Fig. 3:**
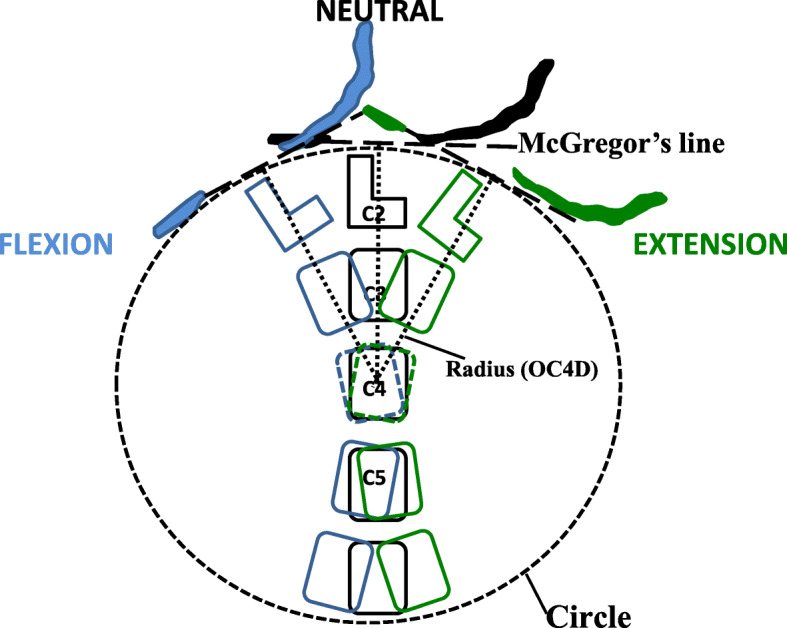
The vertebral body of C4, an apex most visible on radiographs and least affected by the cervical curve, was designated as a landmark. The OC4D can be regarded as the radius of a circle positioned at the center of the C4 vertebral body and tangent to the McGregor’s line

In 1999, Phillips et al. first measured the occiput-cervical distance of OCD by measuring the shortest distance from the most superior aspect of the C2 spinous process to the occipital protuberance in 30 asymptomatic subjects. In this initial study, the value of the OCD in the neutral position was 21.5 ± 1.22 mm, and it was significantly different from OCD values measured in flexion (28.0 ± 1.32 mm) and extension (14.8 ± 1.48 mm) [4]. Seong et al. measured OCDs in 200 normal, sagittal balanced patients (100 male and 100 female patients), and the mean neutral OCD was 22.98 ± 5.10 mm (range, 9.88–38.64 mm), which was significantly different from those in flexion and extension positions [5]. In our present study, the mean neutral, flexion, and extension OCDs were 23.0 ± 4.8 mm, 27.6 ± 6.0 mm and 13.8 ± 4.7 mm, respectively, and we also found that these OCDs were significantly different from one another in neutral, flexion, and extension positions. Unfortunately, correlations between OCD with height, weight, and BMI have not been reported in previous studies. In this study, there was a weak but significant correlation between OCD and height in neutral, flexion, and extension positions, but there was no significant correlation of OCD with weight and BMI. We measured the occiput-cervical distance via the OC4D, and the mean neutral OC4D was found to be 69.0 ± 6.9 mm. Importantly, this neutral OC4D value was not significantly different from those measured in flexion (68.9 ± 6.8 mm) or extension (68.1 ± 6.9 mm). Seong et al. found that the posterior border of C4 serves as a landmark in the apex of cervical lordosis, and that it is therefore the least affected by the cervical curve [5]. We hypothesized that the C4 vertebral body, being the central point of the cervical sequence, is the least affected by motion of the cervical position. Hence, the shortest distance from the center of the C4 vertebral body to the McGregor’s line in each cervical position can be regarded as the radius of a circle positioned at the center of the C4 vertebral body and tangent to the McGregor’s line (Fig. 3). Additionally, in our present study, we found significant positive correlations between OC4D and height, as well as between OC4D and weight, among which OC4D had a stronger correlation with height compared to that with weight. In contrast, the correlation between OC4D and BMI was weak and was not statistically significant. And we also found there was no significant correlation between OC4Ds and OCDs in this study. On the one hand, it is due to the difference of measurement methods. On the other hand, it is more important that variation of C2 spinous process has great influence on the individual difference of measurement results [13].

**Fig. 4:**
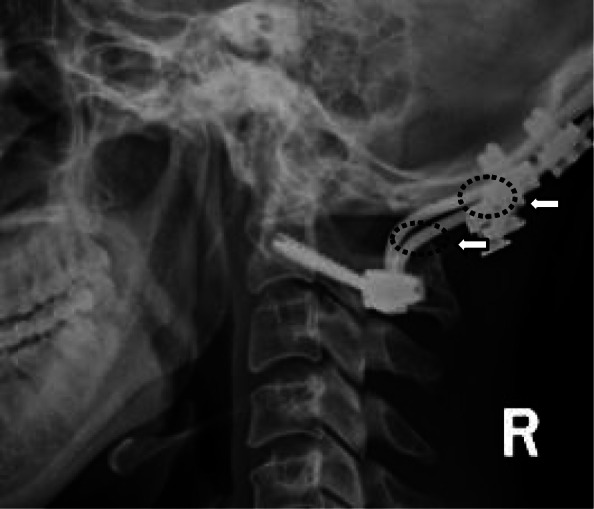
The C2 spinous process and occipital protuberance can be occluded by fixed implants during occipital-cervical fusion (OCF) and the implants could affect the measurements of the occipitocervical distance (OCD)

We found that our novel OC4D measurement has unique advantages compared with those of the OCD in previously reported studies [4, 5, 13]. First, we found that the OC4D was a more accurate parameter compared to OCD in our present study. Additionally, in terms of the OCD, previous studies have demonstrated significant inter-individual morphologic variation in the C2 spinous process (including gender differences). Jiang et al. found that variations in the C2 spinous process may affect the OCD value, and that there was a significant difference in OCD values between male and female subjects [13]. Additionally, the inter- and intra-observer reliabilities of OCDs had ICC values of only 0.651 and 0.754 in a previous study [5]. In the present study, the ICC values of inter- and intra-observer reliabilities for OCDs were moderate to good based on evaluation using standard conventions (see Methods section). We found that the posterior margin of the hard palate, occipital bone, and C4 vertebra (with less bone variation) were clear on lateral radiographs. The ICC values of inter- and intra-observer reliabilities for OC4Ds were more than 0.93 in neutral, flexion, and extension positions, which were significantly higher than those for OCDs. Second, we found that the OC4D was less affected by different positions of the head and neck in neutral, flexion, or extension positions. The alignment of the subaxial spine can influence the occipitocervical alignment required to ensure a functional position of the occiput. However, this variable was not specifically measured in the current study. At present, only a few studies have reported OCD measurements and have shown that neutral OCDs are significantly different from those in flexion and extension positions [4, 5, 10]. In contrast, in our present study, there was no significant difference in the OC4Ds among neutral, flexion, and extension positions (whereas there was for OCDs). This finding may have clinical significance for the use of the OC4D in guiding reduction during operations when the occiput-cervical region is not in a neutral position. Third, the OC4D is not occluded by implants and may therefore represent a valuable intraoperative tool for designing of fusion implants and testing of restoration in the operating room. However, there are no reports showing that the C2 spinous process can be occluded by fixed implants during OCF and that the implants could affect OCD measurements (Fig. 4). Previous literature has stated that it may be difficult to visualize the tip of the dens on radiographs, or that the dens may be absent or fixed in an abnormal position in many conditions under which OCF is performed [14, 15]. Therefore, it may be difficult and inaccurate to evaluate vertical reduction of the occipitocervical region by the distance from the odontoid tip to the McGregor’s line during surgery. Wang et al. first described lower cranial nerve palsy following vertical over-distraction after OCF in four patients who had atlantoaxial dislocation with or without basilar invagination, and the symptoms of all patients were alleviated to different extents by releasing the screw cap and recovery to partial reduction of the occipitoatlantal anatomy [12]. However, our novel OC4D method avoids the occlusion caused by implants and the uncertainty of bony landmarks on radiographs, and therefore may represent a useful tool for estimating and testing the restoration of occipitoatlantal anatomy via regulation of fixed implants.

Limitations of the present study included the demographic data not being matched for age, as well as our sample size being relatively small. In spite of these limitations, our study presented a new method for measurement of the occipital-cervical distance, which may have practical valuable for guiding and testing the restoration condition of the occipital-cervical region. Another limitation of this study is that only cervical spinal radiographs were analyzed, as there were no data regarding the overall sagittal alignment of the spine. Although previous studies have reported that cervical curvature can be affected by overall spinal sagittal imbalance [16–19], only normal subjects with a normal cervical curvature were included in our present study, and we found no difference in OC4Ds as a function of changes in cervical curvature in neutral, flexion, and extension positions. However, we also recognize that cervical curvature changes can accelerate cervical degeneration, which may affect the results of OC4D measurements. Thus, future studies are needed to obtain more reliable measurements regarding cervical and overall spine sagittal alignment parameters, and to further explore the effect of spinal sagittal parameters on the OC4D. In addition, this present study did not provide the OC4D in patients with craniocervical joint instabilities as a clinically relevant comparator. Hence, we will measure the OC4D values of patients with craniocervical malformation as well as analyze the effect of OC4D fixation selection on the clinical efficacy and patient complications during OCF in future research.

## Conclusions

In this study, we proposed and introduced a new OC4D method for estimating the occipital-cervical distance via the shortest distance from the center of the C4 vertebral body to the McGregor’s line (i.e. the OC4D). We found that OC4Ds were not significantly different across neutral, flexion, or extension positions in males or females, and that OC4Ds were significantly positively correlated with both height and weight. Hence, our findings suggest that the OC4D may represent a valuable parameter and intra-operative tool for guiding vertical restoration during OCF for patients with altered occiput-cervical anatomy.

## Data Availability

Data will be available upon request to the first author, TC.

## Abbreviations

OC4D: Occiput-C4 distance
OCD: Occipitocervical distance
BMI: Body mass index
ICCs: Intra-class and inter-class correlation coefficients
OCF: Occipitocervical fusion
